# Infectious Spondylitis Caused by Staphylococcus lugdunensis Following an L-PEN Procedure: A Case Report

**DOI:** 10.7759/cureus.103371

**Published:** 2026-02-10

**Authors:** Youngkwon Yang, Kyungryeol Kang, Eundong Lee, Jeongeun Lee, Seeun Jung

**Affiliations:** 1 Department of Anesthesiology and Pain Medicine, Veterans Health Service Medical Center, Seoul, KOR

**Keywords:** epidural neuroplasty, infectious spondylitis, lower back pain, procedure-related spinal infection, staphylococcus lugdunensis

## Abstract

We report a rare case of infectious spondylitis caused by *Staphylococcus lugdunensis* following lumbar percutaneous epidural neuroplasty (L-PEN). A 78-year-old man with chronic low back pain refractory to conservative treatment underwent L-PEN and subsequently developed worsening back and bilateral hip pain 18 days after the procedure, followed by progressive motor weakness. Initial laboratory evaluation revealed markedly elevated inflammatory markers. Contrast-enhanced magnetic resonance imaging demonstrated diffuse multilevel epidural enhancement with vertebral endplate involvement, consistent with infectious spondylitis. Blood cultures grew oxacillin-susceptible *Staphylococcus lugdunensis*. Empirical intravenous vancomycin was initiated and subsequently de-escalated to targeted β-lactam therapy based on susceptibility results. Despite extensive epidural and paraspinal involvement, no surgically drainable abscess was identified, and the patient was managed conservatively. After a total of 12 weeks of antimicrobial therapy, including intravenous treatment followed by oral step-down therapy, the patient achieved complete clinical and radiological recovery without surgical intervention. This case highlights the pathogenic potential of *Staphylococcus lugdunensis* in procedure-related spinal infections and underscores the importance of early diagnostic imaging and appropriate microbiological evaluation when pain worsens after spinal interventions.

## Introduction

Infectious spondylitis is a disease that affects the vertebral body, intervertebral disc, or surrounding tissues. In South Korea, the incidence of pyogenic spondylodiscitis has increased over the past decade, likely related to population aging, higher prevalence of comorbidities, and the growing number of invasive spinal procedures [1]. 

*Staphylococcus aureus* is the predominant causative agent in pyogenic infectious spondylitis, accounting for approximately 40-60% of cases, followed by *Streptococcus* species and Gram-negative organisms [2,3]. Whereas, coagulase-negative *Staphylococci* (CoNS) species are less commonly implicated and are often considered contaminants. However, unlike other CoNS, *Staphylococcus lugdunensis* exhibits virulence and clinical behavior similar to *S. aureus* in bone and joint infections, and its isolation from blood or sterile sites should be considered clinically significant rather than contamination [4].

Infectious spondylitis may arise from hematogenous spread, direct inoculation, or contiguous extension; in recent years, an increasing proportion of cases has been associated with spinal procedures. These include postoperative infections following spine surgery, infections related to indwelling epidural catheters, and iatrogenic infections after percutaneous spinal interventions such as epidural steroid injections, facet joint procedures, or epidural neuroplasty [3,5]. 

Lumbar percutaneous epidural neuroplasty (L-PEN) is a minimally invasive procedure commonly used to manage chronic low back pain refractory to conservative treatment. Although generally safe, infectious complications following L-PEN are rare but can be severe. We present a rare case of *S. lugdunensis* infectious spondylitis following L-PEN.

## Case presentation

A 78-year-old man with a medical history of Parkinsonism and three-vessel coronary artery disease treated with coronary stent placement presented with chronic low back pain radiating to the right lower extremity, suggestive of lumbar spinal stenosis.

Because of these symptoms, lumbar magnetic resonance imaging (MRI) was performed, which demonstrated multilevel degenerative changes, including spinal stenosis from L1 to S1, degenerative grade I spondylolisthesis at L4/5, retrolisthesis at L1/2 and L2/3, and degenerative spondylosis with scoliosis. There were no radiological findings suggestive of infection.

The patient was initially managed with conservative treatment, including oral medications such as NSAIDs, gabapentinoids, and a 5-HT₂A receptor antagonist (sarpogrelate), in addition to physical therapy. However, his symptoms persisted despite medical management. He subsequently underwent a right L5 transforaminal epidural steroid injection (TFESI), which provided only short-term pain relief lasting approximately one week. An epidurogram performed during TFESI suggested possible adhesions around the right L5 nerve root, corresponding to the patient’s predominant pain in the right L5 dermatome.

Surgical decompression was not pursued at this stage because the patient did not demonstrate objective neurological deficits, and imaging findings were more consistent with moderate rather than severe lumbar spinal stenosis, making conservative and interventional pain management a reasonable initial strategy.

Given the limited and transient benefit of TFESI and the suspicion of epidural adhesions, lumbar percutaneous epidural neuroplasty (L-PEN) was selected as the next-step intervention to mechanically and chemically lyse epidural adhesions and improve medication delivery to the affected nerve root.

The procedure was performed via a caudal approach under fluoroscopic guidance. After standard sterile skin preparation with povidone-iodine and chlorhexidine and placement of a sterile drape, a caudal epidural needle was inserted into the sacral hiatus. A flexible neuroplasty catheter (BioVision catheter) was then advanced to the right L5 nerve root level. After confirming appropriate catheter position with contrast injection and observing diffusion of contrast around the right L5 nerve root, suggesting lysis of suspected adhesions, epidural adhesiolysis was performed. The procedure was completed without immediate complications, and no new neurological deficits were observed; the patient was discharged home the same day.

Approximately 18 days after the procedure, he visited the emergency department due to worsening low back pain and bilateral hip pain. At that time, vital signs were stable, no focal neurological deficits were noted, and no additional laboratory or imaging studies were performed; he was discharged without further evaluation.

**Figure 1:**
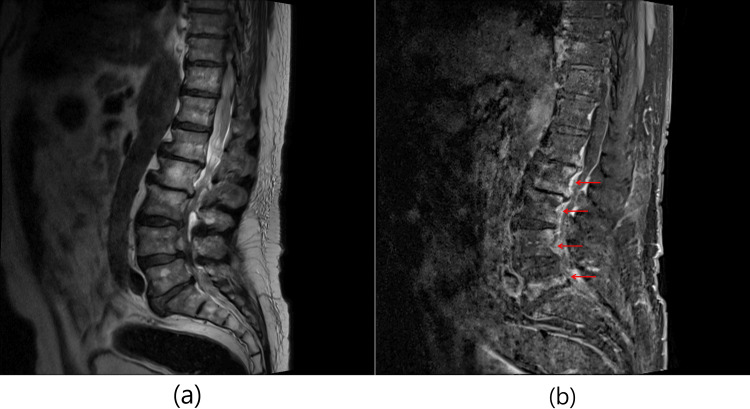
Spine sagittal magnetic resonance imaging (MRI) was obtained at diagnosis. (a) T2-weighted image demonstrates endplate destruction and decreases height of disc space of L5/S1 and thickening along the epidural space below L1 level. (b) Contrast-enhanced T1-weighted image with fat suppression demonstrates subtle bone marrow enhancement at the posterior body adjacent to the spinal canal of L2, L3, and L4 and enhancement along the epidural space below the L1 level (red arrow).

Persistent pain and newly developed motor weakness prompted reevaluation in the outpatient clinic, and he was admitted for further workup (hospital day 0; approximately 21 days after L-PEN). A comparison of pre- and post-procedural symptoms is summarized in Table 1. Contrast-enhanced thoracolumbar MRI revealed diffuse thickening and enhancement of the epidural space below the L1 level, resulting in aggravated central canal compression from L2 to S1. Additional findings included subtle bone marrow enhancement of the posterior vertebral bodies at L2-L4, newly developed bilateral psoas muscle swelling with enhancement at the L4-L5 level, and endplate destruction with decreased disc height at L5/S1, consistent with infectious spondylitis (Figure 1).

**Table 1:** Comparison of pre- and post-procedural symptoms. *Post-procedural symptoms were evaluated at the time of the patient’s visit to the pain clinic, when worsening pain and new motor weakness raised suspicion for infectious spondylitis and prompted contrast-enhanced MRI.

	Back pain (NRS)	Radiating pain	Neurologic deficit
Pre-procedural	6-7	Right lower extremity (L5 dermatome)	Absent
Post-procedural*	8	Bilateral hip/lower extremity pain	Bilateral lower-extremity motor weakness (grade IV)

Initial laboratory evaluation demonstrated an erythrocyte sedimentation rate (ESR) of 58 mm/hr and a markedly elevated C-reactive protein (CRP) level of 125.78 mg/L. The white blood cell (WBC) count was 6.95 × 10³/μL, and a low-grade fever was noted. Blood cultures were obtained, and empirical intravenous vancomycin was initiated to cover potential healthcare-associated pathogens, including methicillin-resistant *Staphylococcus aureus*.

On hospital day five, blood cultures obtained on hospital day one grew oxacillin-susceptible *Staphylococcus lugdunensis*. Repeat blood cultures were obtained on hospital day three, and the final report, available on hospital day seven, showed negative results, indicating clearance of bacteremia within three days of hospitalization. A transthoracic echocardiogram (TTE) was planned if bacteremia persisted; however, because follow-up blood cultures became negative by hospital day three, TTE was not performed.

**Figure 2:**
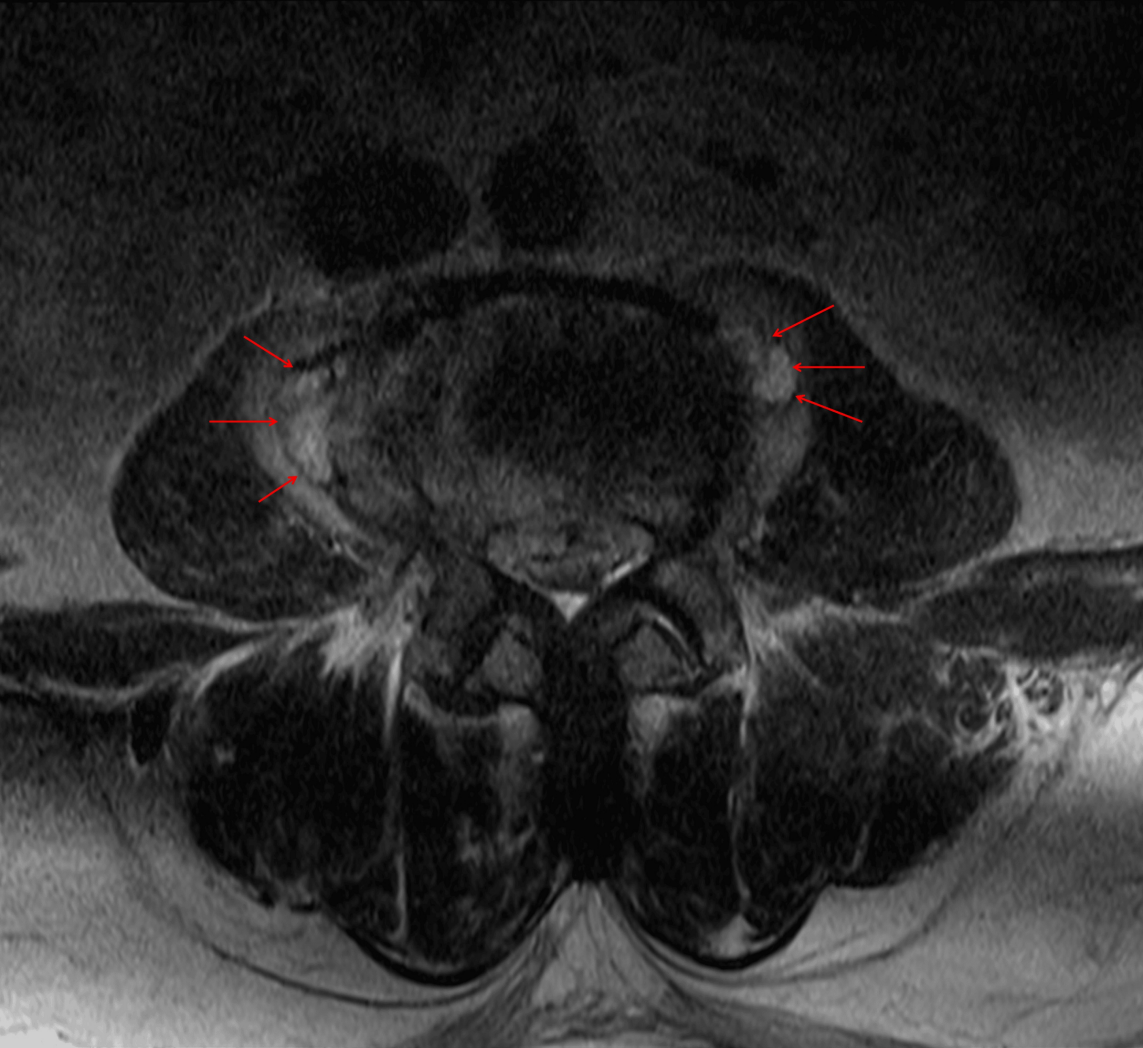
Spine axial magnetic resonance imaging (MRI) was obtained in the second week of hospitalization. A T2-weighted image shows several newly noted abscess pockets in bilateral psoas muscles at L4 levels.

Once susceptibility testing confirmed oxacillin-susceptible *S. lugdunensis*, antimicrobial therapy was de-escalated to intravenous cefazolin (2 g every 8 h). Although inflammatory markers initially decreased, a mild re-elevation of CRP was observed during the second week of hospitalization. Given the extensive epidural and paraspinal involvement on imaging (Figure 2) and the need for optimal tissue penetration, antimicrobial therapy was switched to intravenous nafcillin. Neurosurgical consultation did not identify a surgically drainable abscess, and conservative management was recommended.

By approximately hospital week seven, inflammatory markers were trending downward but had not yet normalized. Accordingly, we elected to maintain intravenous antimicrobial therapy until complete normalization of inflammatory markers was achieved, given the extensive epidural and paraspinal involvement on MRI, the patient’s advanced age with significant comorbidities, and the known aggressive behavior of *Staphylococcus lugdunensis* in deep-seated infections. In addition, definitive surgical source control was not feasible because no drainable abscess was identified.

By approximately hospital week 10, inflammatory markers had fully normalized, and the patient demonstrated marked clinical improvement. He was therefore transitioned to oral ciprofloxacin (750 mg twice daily) plus rifampin (300 mg twice daily) as step-down therapy to complete a planned total treatment duration of 12 weeks. The patient was discharged, and subsequent outpatient laboratory studies confirmed sustained normalization of inflammatory markers, allowing discontinuation of antimicrobial therapy.

The overall clinical course is summarized in Table 2.

**Table 2:** Summary of clinical and microbiological course. Representative ESR and CRP values, changes in antimicrobial therapy, and serial blood culture results throughout the treatment period are presented.

HD day	ESR (mm/hr)	CRP (mg/L)	Antibiotics	Blood culture
On admission	58	125.7	Vancomycin	S. lugdunensis
Day 05	66	69.1	Cefazolin	negative
Day 07	73	51.9	Cefazolin	negative
Day 09	70	35.8	Cefazolin	-
Day 17	73	31.3	Cefazolin	-
Day 21	74	38.4	Nafcillin	-
Day 49	51	22.1	Nafcillin	
Day 72	normal	normal	Ciprofloxacin+ rifampin(PO)	-
Day 84	normal	normal	stop	-

## Discussion

This case demonstrates a rare instance of infectious spondylitis caused by *S. lugdunensis* following lumbar percutaneous epidural neuroplasty. Although *S. lugdunensis* is a coagulase-negative *staphylococcus* and a normal skin commensal that preferentially colonizes the perineal region, it exhibits virulence characteristics more akin to *Staphylococcus aureus*, with the potential to cause invasive and destructive infections [6-8].

Infectious spondylitis due to *S. lugdunensis* has been infrequently reported; however, when present, it is often associated with aggressive disease features such as extensive vertebral body involvement, epidural extension, and paraspinal or psoas abscess formation [4,9]. These features are consistent with the organism’s *S. aureus*-like pathogenic potential and underscore its clinical significance when isolated in invasive spinal infections.

Although the exact source of bacterial contamination could not be definitively identified, the temporal relationship between L-PEN and symptom onset suggests a procedure-related infection. Possible mechanisms include (1) skin flora inoculation during needle or catheter insertion despite standard sterile precautions, (2) retrograde bacterial migration along the neuroplasty catheter, or (3) hematogenous seeding of the epidural space facilitated by tissue disruption from adhesiolysis. Given that *S. lugdunensis* is a known skin commensal with a predilection for invasive infection, contamination from skin flora during the procedure is a plausible source [6,7].

Spinal infections following interventional pain procedures are uncommon, yet their diagnosis is frequently delayed because the initial clinical presentation is often insidious and nonspecific. Although infectious spondylitis is classically described by a triad of back pain, fever, and neurological deficits, this complete triad is observed in only a minority of patients. Previous studies have shown that while back pain is present in most cases, fever occurs in approximately 35-60% and neurological deficits in fewer than 15-20%, with fewer than 10% of patients exhibiting all three features at initial presentation [10]. Consequently, infectious spondylitis may be easily overlooked in its early stages, particularly when neurological findings are absent.

This diagnostic challenge highlights the importance of maintaining a high index of suspicion for infectious complications when back pain worsens or fails to improve after spinal interventions, especially in elderly patients with comorbidities, in whom delayed diagnosis has been associated with worse clinical outcomes. Early diagnostic imaging and appropriate microbiological evaluation are therefore critical to prevent disease progression and irreversible complications.

Importantly, *Staphylococcus lugdunensis* should be recognized as a true pathogen in this clinical context, even when isolated from blood cultures as a coagulase-negative *staphylococcus* [11,12]. The organism has been strongly associated with deep-seated infections and sustained bacteremia, and persistently positive blood cultures for *S. lugdunensis* in patients with suspected infectious spondylitis may, in selected cases, obviate the need for image-guided biopsy when clinical, laboratory, and radiologic findings are concordant [13].

Therapeutic strategies for *S. lugdunensis* infections generally parallel those used for methicillin-susceptible *S. aureus*. Most isolates remain susceptible to antistaphylococcal β-lactam antibiotics such as cefazolin or oxacillin, which are considered first-line therapy and are preferred over glycopeptides because of their superior bactericidal activity [12,14]. Current guidelines recommend a minimum of six weeks of antimicrobial therapy for infectious spondylitis; however, prolonged treatment durations may be warranted in cases with extensive epidural or paraspinal involvement, delayed clinical response, or infection caused by highly virulent organisms [12].

In the present case, a 12-week course of targeted antimicrobial therapy resulted in complete clinical and radiological recovery without the need for surgical intervention, illustrating that favorable outcomes can be achieved with timely recognition and prolonged medical management.

While intravenous antibiotic therapy remains the standard initial approach for infectious spondylitis, an early transition to oral antibiotics may be considered in carefully selected patients who demonstrate clinical stability, resolution of systemic symptoms, decreasing inflammatory markers, and adequate control of epidural or paraspinal infection. In such circumstances, oral step-down therapy should be limited to agents with high bioavailability and proven activity against the identified pathogen, including fluoroquinolones (with or without rifampin), linezolid, or oral β-lactams such as dicloxacillin or amoxicillin-clavulanate for methicillin-susceptible staphylococcal infections [15-17].

## Conclusions

*Staphylococcus lugdunensis* should be regarded as a clinically significant pathogen in patients with suspected spinal infections, even when isolated from blood cultures as a coagulase-negative *Staphylococcus*. This case emphasizes the importance of early diagnostic imaging, appropriate microbiological evaluation, and prolonged targeted antimicrobial therapy in the management of procedure-related infectious spondylitis. 

Early diagnosis is essential, as delayed recognition of infectious spondylitis has been associated with increased rates of neurological complications, the need for surgical management, and prolonged hospitalization, whereas timely diagnosis allows for earlier initiation of targeted antimicrobial therapy and may prevent irreversible structural or neurological damage.

In cases with extensive epidural or paraspinal involvement, or when central nervous system (CNS) extension is a concern, the choice of definitive β-lactam therapy merits careful consideration; although cefazolin is effective for methicillin-susceptible *S. lugdunensis*, anti-staphylococcal penicillins such as nafcillin or cloxacillin may be preferred when optimal CNS penetration is desired.
